# Transcranial magnetic stimulation of the left middle frontal gyrus modulates the information people communicate in different social contexts

**DOI:** 10.1038/s41598-023-36192-3

**Published:** 2023-06-20

**Authors:** Beatriz Martín-Luengo, Alicia Nunez Vorobiova, Matteo Feurra, Andriy Myachykov, Yury Shtyrov

**Affiliations:** 1 Centre for Cognition and Decision Making, Institute for Cognitive Neuroscience, HSE University, Moscow, Russia 101000; 2 Department of Psychology, Northumbria University, Newcastle upon Tyne, UK; 3 Department of Clinical Medicine, Center of Functionally Integrative Neuroscience (CFIN), Aarhus University, Aarhus, Denmark

**Keywords:** Neuroscience, Cognitive neuroscience

## Abstract

Neocortical structures of the left frontal lobe, middle frontal gyrus (MFG) in particular, have been suggested to be linked to the processing of punishing and unpleasant outcomes in decision tasks. To assess the role of left MFG (lMFG) in communicative decisions, we used repetitive transcranial magnetic stimulation (rTMS) to inhibit its function during communicational exchanges under two types of social contexts: formal and informal. Three groups of participants received an offline 1-Hz inhibitory rTMS of lMFG, right MFG as an active control site, or lMFG sham/placebo TMS as a passive control condition. Participants’ task included answering difficult general-knowledge questions, rating their confidence in their answers’ correctness, and, finally, deciding if they would report or withhold these answers in formal and informal social contexts. There were significantly more reported than withheld answers in the informal context in all groups. The formal context showed no differences between reported and withheld answers in both control conditions, while, crucially, real rTMS of lMFG produced a different pattern, with more withheld than reported answers. Thus, lMFG inhibition seems to result in more rational decisions made only in formal communication contexts, where there is a perception of a certain pressure or possible negative outcomes. In informal social contexts and in the absence of negative consequences the pattern of answers did not change, regardless of the reporting strategy or the TMS protocol used. These results suggest selective context-dependent involvement of the lMFG in decision-making processes during communicational exchanges taking place under social pressure.

As a rule, people do not share information with others indiscriminately^1^, and our behavioural patterns in communicating information vary considerably depending on the social context or on the impression we want to make^2–4^. These features are socially grounded, and they are learned through our life experience. Neural underpinnings of this behavioural flexibility during communicational exchanges, however, remain largely unexplored. The brain bases of social communication are studied within a young interdisciplinary field called neuropragmatics, with relatively little work done so far using approaches beyond those common in psycho- and neuro-linguistic studies^5^. In this study, we aimed to investigate the neural substrates supporting information exchange during a conversation held in different social contexts.

One common mode of interpersonal communication, which usually serves as the basis for having a conversation between two or more individuals, is asking and answering questions. In such a communication, we normally have to consider our own knowledge about the conversation topic as well as what we believe to be the expectations of our interlocutor. This, in turn, determines the type of answers we provide to the interlocutor’s questions as well as our willingness to provide them or, vice versa, refrain from sharing information^6^. The mechanisms underpinning these communicational decisions are still under debate and have only recently become a subject of experimental scrutiny. For example, in a recent study^3^, participants answered difficult general-knowledge questions before deciding whether to report or to withhold their answers depending on a given social context. The main goal was to investigate the social context effect on sharing information whose correctness is uncertain to participants. Following the *dual-criterion satisficing model*^7–9^, participants were expected to comply with two criteria when deciding to report an answer: (1) enough *confidence in the correctness* of their answers, and (2) a certain level of *informativeness*, i.e., whether their answers are informative enough (e.g., we can be confident that “Mount Everest is higher than 1000 m” is a correct response, but at the same time it is not sufficiently informative regarding Everest’s actual height). The results showed that participants varied the amount of reported and withheld answers depending on the given *social context*, that is, their communicational strategy considered not only the two above criteria but also the perceived *incentive structure* of the particular context^8^. For example, participants were willing to report almost all their answers in an informal context (e.g., talking with friends) regardless of their confidence in the answers’ correctness. So, the incentive structure of being in an informal social situation seems to elicit a tendency to provide any information for the sake of being communicative. When, however, the context was formal (a job interview), the number of withheld answers increased. Furthermore, in the latter situation the participants preferred simpler, less ambiguous answers, presumably to achieve a better impression of their certainty/confidence in the provided information. Still, although all the questions were rather difficult, participants were willing to report a certain number of answers, likely because not providing any answer (as a strategy to keep overall accuracy high) may be not socially acceptable in a more formal setting^7^. From a theoretical viewpoint, this study demonstrated that under uncertainty, participants apply a different *memory reporting strategy* depending on the incentive structure elicited by the specific social context in which the information exchange takes place.

To study the neural mechanisms of such strategic meta-regulation of memory reporting, some studies used functional magnetic resonance imaging (fMRI). Importantly, an fMRI experiment, based on earlier seminal behavioural work^9^, pointed to the involvement of the left middle frontal gyrus (lMFG), among others brain areas, in the control processes^10^ (report vs. withhold; see Table 3A in Risius et al.^10^). Participants were first presented with a video outside the scanner and later, inside the scanner, they read true and false sentences related to the video. Participant’s primary task was to decide whether the sentences were correct or not while the secondary task was to rate the confidence in their selection. They finally had to decide whether they would report their selected answer or not. Importantly, participants had a monetary compensation for reported correct answers, that is, participants were encouraged to be accurate. Deciding whether to report or withhold their answer activated the lMFG, while the memory and metamemory tasks (correctness judgement and confidence rating) did not, clearly suggesting lMFG’s role in the control process taking place within a specific context, rather than in memory/retrieval per se.

Furthermore, a body of converging evidence regarding strategic decision-making, obtained in patients with bilateral frontal lobe lesions^11^ as well as healthy individuals^12^, points towards the involvement of the lMFG as one of the brain areas underlying the processing of punishing and/or unpleasant outcomes. This has been shown, for example, by research on retrieval of semantic information^13^ as well as on gambling^12^. The lMFG has been also shown to play a critical role in studies investigating executive functions, specifically response inhibition^14–16^. For instance, Collete et al.^14,15^ used a sentence completion design whereby the suggested word would fit the sentence in one of the two conditions (response initiation) but not in the other one (response inhibition). Using positron emission tomography (PET), researchers found that the response inhibition condition increased the activation of the left prefrontal areas including the middle frontal gyrus. Executive functions, particularly inhibition, play a significant role in social communication. Indeed, disorders such as ADHD and Tourette syndrome are accompanied by both neuroanatomical changes in the middle frontal gyrus and poor communication abilities attributed to disrupted inhibitory function^17,18^. Furthermore, social communicative deficits such as socially inappropriate responses, impulsive answering, or perseveration on a topic have been reported in individuals with traumatic brain injuries that affect brain areas involved in inhibitory control^19,20^. To summarise, existing evidence suggests that the lMFG is involved in withholding answers^10^ as well as in evaluating a certain pressure, punishment or negative outcome^12^, and is related to deficits in social communication resulting from impairments in the inhibitory mechanisms^14–18^.

Furthermore, research on neural activation while performing a neuroeconomic task also pointed to the recruitment of frontal cortices areas in the decision-making process. This is particularly the case when the so-called System 2 is used^2,21^. In decision-making research, System 1 is related to automatic no-deliberative decision process, whereas System 2 is defined as the one in charge of decisional processes where an active deliberation of the options is made. Translated to the present research and the results reviewed above^3^, participants' behaviour in an informal context might rely on System 1 since there is no pressure or punishment for any particular behaviour. However, the formal context should engage System 2 since participants will need to take into consideration what would be expected from them and, therefore, carefully consider the options available, their performance in previous trials in the same condition, etc. Thus, inhibiting lMFG function may selectively impair communicative decisions made in such a context, whilst not affecting other social situations.

Importantly, however, previous fMRI results were obtained without manipulating the stimulus’ context and thus cannot provide evidence on the interaction between the social context and the communicative memory-control processes. Furthermore, fMRI as well as other mainstream neuroimaging methods (such as EEG or MEG) can only offer correlational evidence but cannot directly speak to the causal relationship between the activated areas and the cognitive phenomena at hand. Establishing such a relationship would require a different approach, such as using brain stimulation techniques that can modulate the functional state of an area which can in turn affect behavioural outcomes. The present study was aimed at filling this gap. Following up on the above evidence from the behavioural studies of context effects on communicational exchanges and the fMRI studies regarding the involvement of lMFG in control processes of memory reporting and punishment, we set out to explore the involvement of lMFG in the perceived reward-punishment system in social interactions. To that end, we used transcranial magnetic stimulation (TMS), a non-invasive brain stimulation technique providing a causal window onto the relationship between specific brain regions and their cognitive functions. Repetitive TMS (rTMS) induces transient inhibition in the stimulated area, lasting from 30 to 60 minutes^22^. Following the previous studies on communicational exchanges during social interactions, we decided to use two social contexts, a formal and an informal one. Moreover, we employed difficult general-knowledge questions which have been shown to be a useful tool to study regulatory processes in sharing information and, importantly, have been normed for a number of parameters. We hypothesised that a transient inhibition of lMFG would result in an interruption of the perceived negative outcome. This implies a selective influence on withholding answers specifically in the formal context, where giving incorrect information or presenting oneself as less knowledgeable may have negative consequences for the individual. This putative inhibitory effect of rTMS may ease the pressure to provide a certain amount of information, but as the questions are difficult, will result in a decrement in the number of reported answers in the formal context specifically. On the other hand, in the informal context we expected our participants to keep providing most of their answers regardless of TMS employment, since no punishment or any type of pressure should be perceived in such a context. To control for unspecific TMS effects, we used a between-group design and, in addition to target lMFG rTMS group, employed two control groups: an active rTMS control group who received right-hemispheric MFG (rMFG) stimulation, and a passive control group subjected to lMFG placebo (so-called sham) condition imitating the TMS procedure without the actual magnetic stimulation delivered.

## Method

### Participants

**Figure 1:**
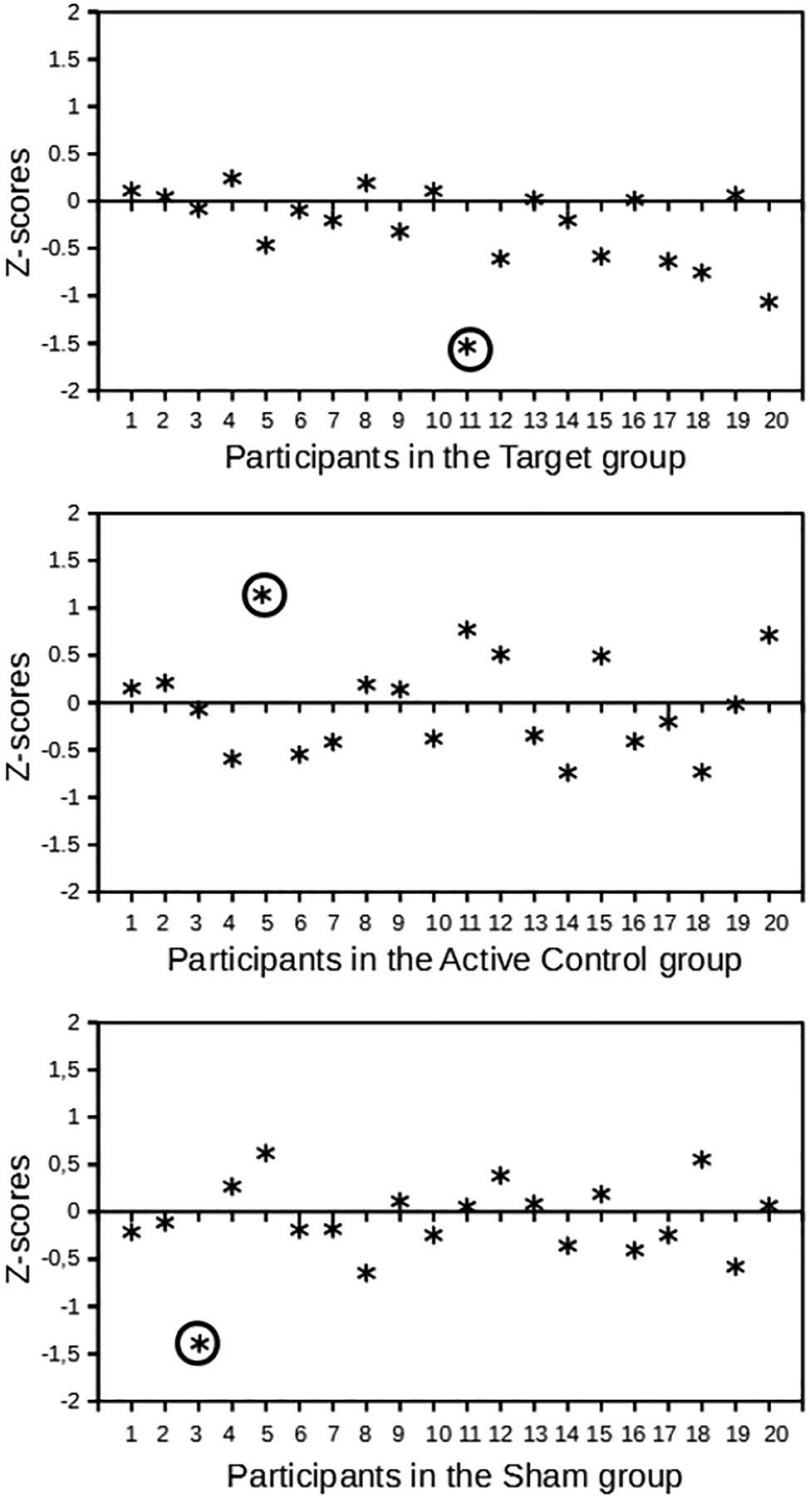
Proportions of reported answers (z-scored) for each participant split by stimulation group. Circled asterisks highlight outliers removed from further analyses.

Sixty volunteers, mostly university students, recruited on social media took part in this experiment in exchange for a small monetary compensation. Before the experiment, all participants gave their written informed consent. Participants were pseudo-randomly assigned to three experimental groups with twenty participants in each (Target group: rTMS of lMFG, Active Control: rTMS of rMFG, Sham: lMFG sham/placebo stimulation). The groups were balanced for gender (14 female and 6 male participants in each) and age (mean age in Target group M = 21.95 years old, *SD* = 3.69; Active Control group M = 23.5 years old, *SD* = 2.78; Sham group M = 23.2 years old, *SD* = 2.93; there were no significant age differences between any of the groups). Here, we report data from fifty-seven participants (n = 19 in each experimental group) after the exclusion of outliers based on their performance (outside the z-score ± 1.5 interval). See Fig. 1 for their distribution. After being detected as outliers because of their poor performance (measured as proportions of reported answers), the answers of three of the participants (one in each group) were checked and found that they did not comply with the instructions (i.e., providing random answers, always selecting only one of the options, giving inflated confidence ratings, etc.).

### Materials

In this type of research on memory reporting it is very important to use materials that could avoid ceiling effects. If the questions were easy, participants would likely choose to report their answers in most cases rendering the manipulation of the report/withhold ratio useless. That is, it is necessary to use questions which, whilst being difficult, are not impossible to answer. To this end, we used a database of 500 general-knowledge questions, previously validated in a sample of participants similar to our sample in terms of age and educational attainment^2,4^. From this database, we selected 40 difficult questions that have been shown to yield answers with low accuracy. Thus, we employed questions, for which participants could generally provide some answers, although they might not be highly confident in their correctness.

### Ethical approval

This work received the approval of the HSE University research ethics committee which guarantees that participants were treated according to the Declaration of Helsinki.

## Procedure

**Figure 2:**
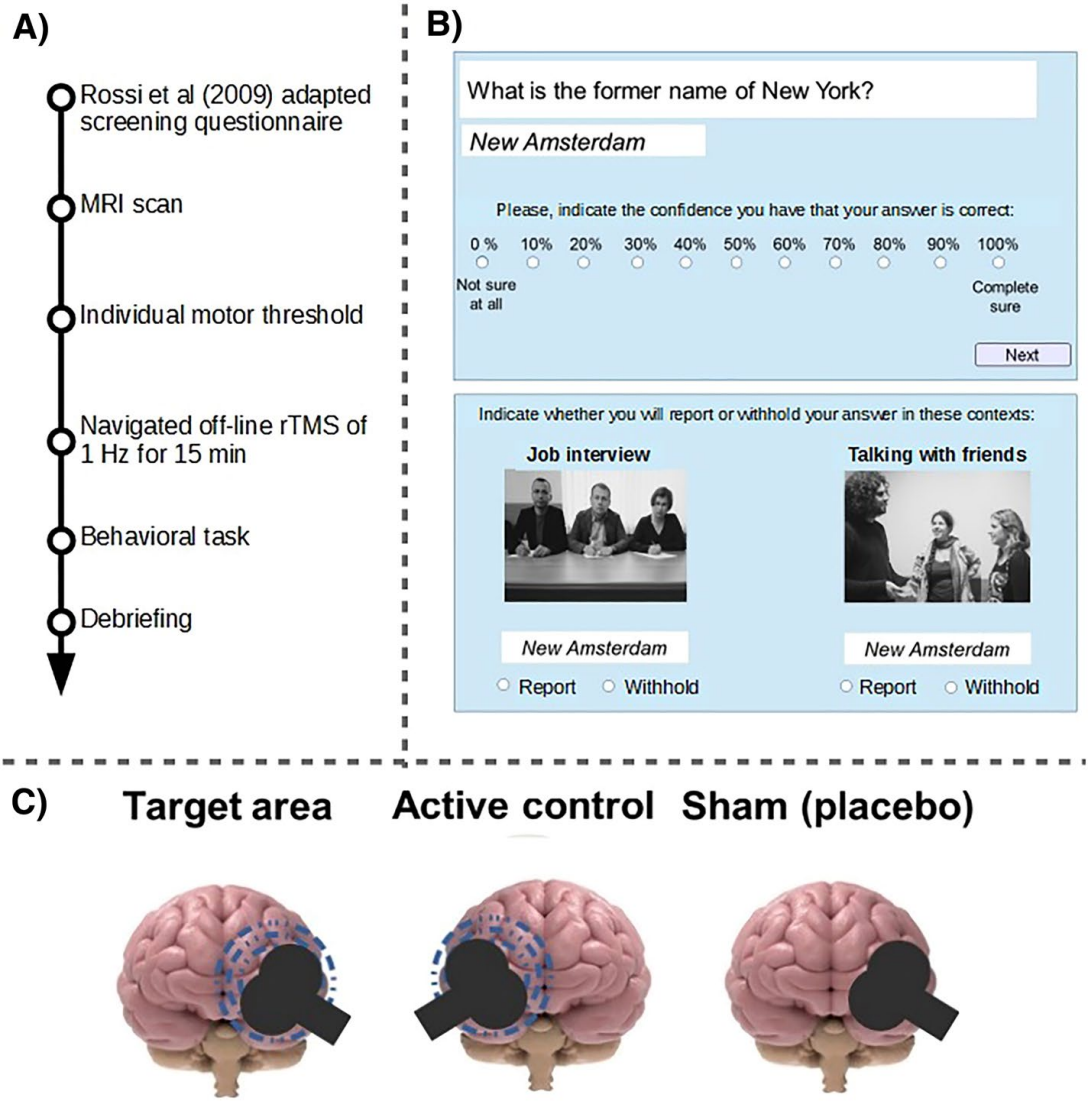
(**A**) Sequence of events in the experimental procedure. (**B**) Illustration of a typical trial sequence. First, participants typed the answer and rated the confidence they had in its correctness. Second, participants chose, for both contexts, if they would report or not the answer they provided before. (**C**) TMS stimulation sites for each group.

See Fig. 2 for experimental procedures, behavioural trial sequence, and TMS stimulation sites.

### TMS implementation

Before taking part in the experiment, participants completed the screening questionnaire^22^ to avoid any potential risks associated with rTMS. On the day before the experiment, participants were requested to avoid a higher than usual intake of caffeine, avoid alcohol, and to have a normal night sleep. Once in the lab, participants were informed about the general procedure of the experiment and requested to read and sign the informed consent form. Next, their individual resting motor threshold (MT) was measured. Being an indicator of relative cortical excitability, MT was used to ensure a similar level of stimulation, with individualised intensity across participants. Single pulses of TMS were applied to the hand area of primary motor cortex (M1) to find the optimal motor hotspot, starting from 45% of stimulator intensity. Motor evoked potentials (MEPs) were recorded using electromyography from the *first dorsal interosseus* (FDI) muscle with disposable adhesive surface electrodes (EB Neuro S.p.A., Florence, Italy). Resting MT was calculated as the minimum intensity required to elicit MEPs of at least 50 µV peak-to-peak amplitude in at least 50% of 10 trials over the hotspot^23^. Once the MT was found, we implemented an off-line 1 Hz rTMS at an intensity of 100% of the MT for 15 min in the Target or Control areas (see below). Sham condition mimicked the procedure of lMFG stimulation without delivering actual TMS, which was achieved by flipping the coil. During the stimulation the coil was held in place manually, visually guided by the individualised hotspot displayed on the computer screen. Duration of 15 min was necessary to provide after-effects lasting at least 30 min on average^24^.

The MNI coordinates for lMFG (x = -22, y = 48, z = 26) were taken from a previous fMRI study (see Table 3(A) in Risius et al.^10^) where a highly similar adaptation of the report option task was used. These coordinates are also compatible with those found in the gambling studies mentioned above^12^. Symmetrical contralateral site (x = 22, y = 48, z = 26) was used for rMFG. Stimulation site was identified for each participant individually using individual T1-weighted structural MR image of each participant’s head and transforming MNI coordinates of MFG to the participant’s native brain space using SPM8 software (UCL Functional Imaging Laboratory; https://www.fil.ion.ucl.ac.uk/spm/software/spm8).

Stimulation was delivered using a butterfly-shape Cool-B-65 coil (MagVenture A/S, Farum, Denmark) powered by the MagPro X100 stimulator (MagVenture). Localite TMS Navigator system (Localite GmbH, Bonn, Germany) was used to ensure the optimal coil positioning based on the individual MR image.

### Behavioural task

Immediately after the rTMS session, participants performed the main behavioural task, during which they were requested to type an answer to a general-knowledge question (40 questions in total), along with their confidence ratings indicating subjective assessment of the correctness of the answer on an eleven-point confidence scale (from 0% ‘not sure at all’ to 100% ‘completely sure’, in 10% steps). Next, their provided answer appeared on a new screen along with two images depicting the suggested social contexts: (1) a formal context in the form of a job interview and (2) an informal context (chatting with friends). Participants were required to indicate if they would report or withhold the previously given answer in each of the two contexts (Fig. 2B). This task was implemented using LiveCode software version 7.1.3 (LiveCode Ltd, Edinburgh, Scotland).

Finally, participants were requested to fill in a questionnaire about their sensations during the TMS implementation procedure. Participants were then thanked and debriefed about the main objective of the experiment. The actual behavioural task lasted around 20 min on average, with the total time of experimental session being approximately 1 h.

### Design and data analysis

A factorial mixed design was used with the Stimulation Group factor (3 levels: Target, Active Control, Sham) manipulated between participants and the Social Context factor (2 levels: Formal vs. Informal)—within participants. Dependent variables were (1) the proportion of reported and withheld answers (report option), (2) the confidence in the correctness of the answer, and (3) response accuracy.

We performed one-way and mixed ANOVAs, which were followed up with two-tailed pairwise comparisons using *Student's t*-tests. Only the reported answers, as well as the confidence ratings for the reported answers, were analysed to avoid a collinearity violation since the report/withhold options are linear transformations of each other^3,4,17^ (i.e., an answer selected as “report” cannot be selected as “withhold”). The level of significance was set at 0.05. We also calculated *Cohen's d* as a measure of effect size and 95% confidence intervals. Goodman–Kruskal gamma correlation (gamma) were also computed as a resolution index to study the degree of confidence ratings distinguishing between correct and incorrect answers. Gamma correlation reflects the degree of the confidence ratings operationalised between correct and incorrect answers^25^. The lack of differences in gamma across groups would indicate that any between-group difference in correct/incorrect ratio of reported answers is not due to differences in the confidence evaluations. The levels and conditions used are specified for each analysis separately.

## Results

The maximum possible number of answers in each group was 760 (40 questions × 19 participants). The actual numbers varied since (a) participants were not always able to provide answers to all the questions and (b) the answers rated with 100% confidence were excluded from the analyses. Regarding the latter, existing research using the report option approach shows that 100% confidence-rated answers (so-called *direct-access answers*) are regulated differently compared to the answers with non-maximum levels of confidence where there is room for regulation, that is, where participants can consider whether the answer is correct or not. These regulatory processes are considered unnecessary for direct-access answers, because participants’ ratings indicate their firm belief in the selected answer^26^. The final analyses included similar numbers of answers across all experimental groups: Target (485), Active Control (466), and Sham (481), without significant between-group differences.

### Accuracy

Before analysing the answers’ distribution, we ruled out the possibility that our data were influenced by the level of the participants’ general knowledge in each group. To this end, a one-way ANOVA with Stimulation as a between-group factor and the answer accuracy as the dependent variable was implement, revealing no significant differences in the participants’ knowledge as a function of Group (Target: *M* = 0.15, *SD* = 0.06, *CI* [0.12, 0.18]; Active Control: *M* = 0.15, *SD* = 0.07, *CI* [0.12, 0.18]; Sham: *M* = 0.15, *SD* = 0.06, *CI* [0.12, 0.18]; *F*(2, 54) = 0.030, *p* = 0.971. *η*_*p*_^*2*^ = 0.001).

### General confidence

Regardless of their knowledge, participants’ confidence in information veracity is a key factor in deciding whether or not to report an answer. If we are highly confident that Sydney is the capital of Australia, we will report that answer irrespective of the context, in which the conversation takes place. Existing research supports the idea that, overall, people tend to be overconfident^27,28^. To assure that performance in each group was similar in this regard, we performed a one-way ANOVA on the confidence levels with Stimulation as a between-group factor, which showed no significant differences between the groups (Target: *M* = 21.55, *SD* = 9.00, *CI* [16.9, 25.1]; Active Control: *M* = 23.64, *SD* = 6.95, *CI* [20.3, 25.7]; Sham: *M* = 23.96, *SD* = 6.51, *CI* [20.3, 25.7]; *F*(2, 27) = 0.566, *p* = 0.571, *η*_*p*_^*2*^ = 0.021).

### Proportions

**Figure 3:**
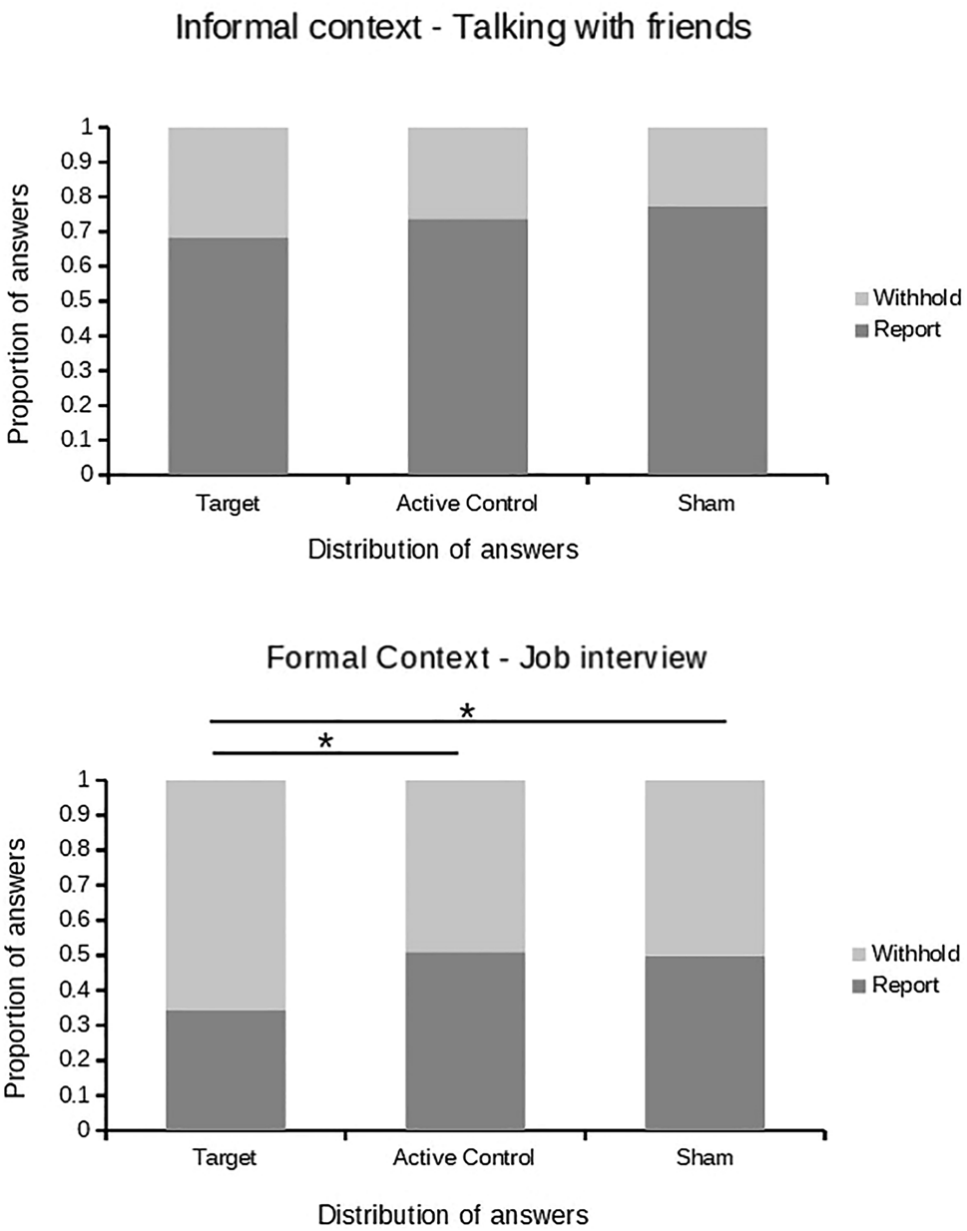
Proportion of reported and withheld answers in each stimulation group (Target lMFG, Active Control, Sham) split by social context (informal, formal) and report option (report, withhold).

Figure 3 illustrates distribution of the answers across groups, contexts, and report options. First, we compared both control groups, Active Control and Sham, and in the absence of differences, *t*(36) = − 0.228, *p* = 0.821, *Cohen’s d* = − 0.074, we further used Active Control only. Then, we conducted a 2 Stimulation Group (Target, Active Control) X 2 Social Context (Formal, Informal) mixed ANOVA on the proportion of reported answers being Stimulation group between and Social Context within-subjects.

We found significant main effects of Social Context (*F*(1,36) = 215.6, *p* > 0.001, η_p_^2^ = 0.417), Stimulation Group (*F*(1,36) = 5.31, *p* = 0.027, η_p_^2^ = 0.065), and their interaction (*F*(1,36) = 4.95, *p* = 0.032, η_p_^2^ = 0.010). Whereas there were significantly more reported answers in the informal than in the formal context in both Stimulation groups (for Target, *M*_*informal*_ = 0.68, *SD* = 0.21, *M*_*formal*_ = 0.34, *SD* = 0.18, *t*(18) = − 11.02,* p* < 0.001, *Cohen’s d* = − 2.528; for Active Control, *M*_*informal*_ = 0.75, *SD* = 0.12, *M*_*formal*_ = 0.50, *SD* = 0.15, *t*(18) = − 9.71,* p* < 0.001, *Cohen’s d* = − 2.228), the proportion of reported answers in the Active Control group for the formal context was higher than for their equivalent in the Target group (*M* = 0.50, *SD* = 0.15 vs. *M* = 0.34, *SD* = 0.18, *t*(36) = − 2.975,* p* = 0.005, *Cohen’s d* = − 0.965), and no differences in the informal context on this comparison (*M* = 0.75, *SD* = 0.12, vs. *M* = 0.68, *SD* = 0.21, *t*(36) = − 1.313,* p* = 0.187, *Cohen’s d* = − 0.426).

### Confidence in the reported answers

**Figure 4:**
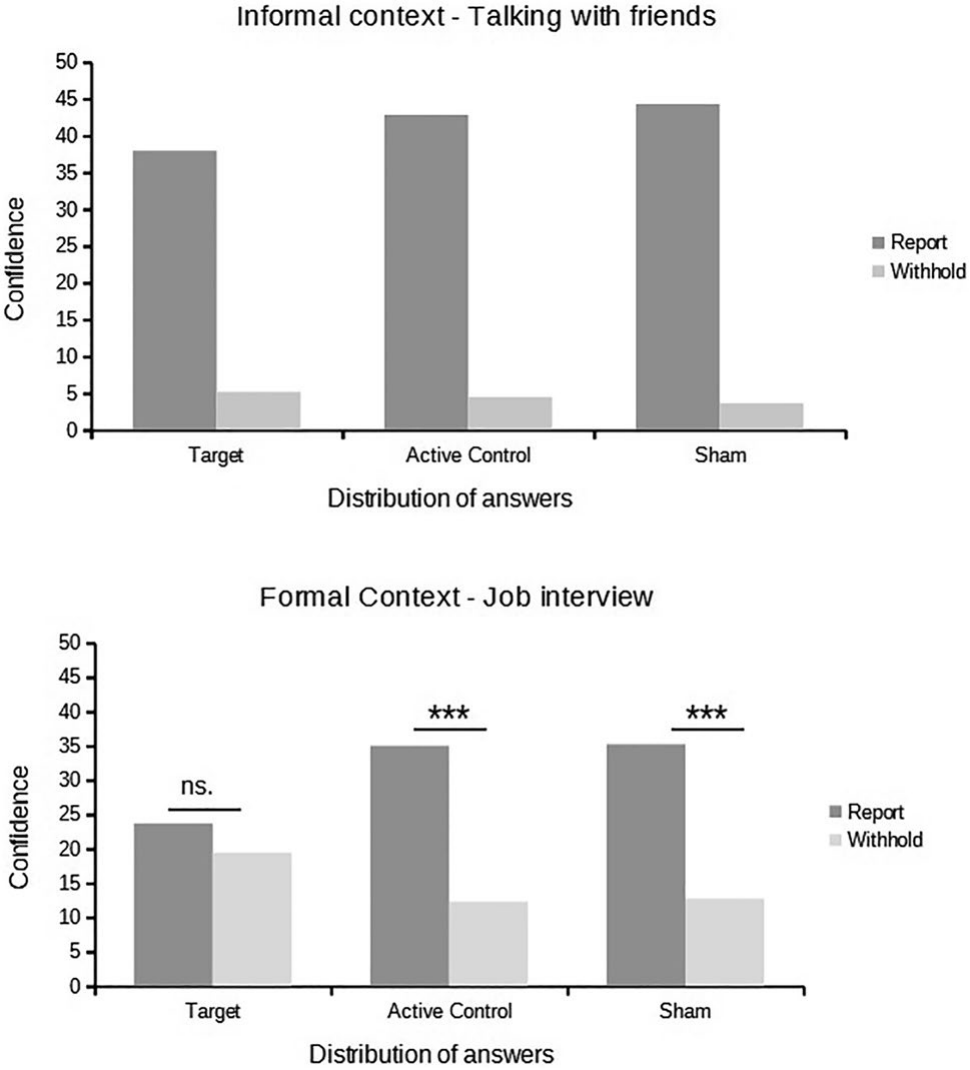
Confidence ratings for reported and withheld answers in each stimulation group (Target lMFG, Active Control, Sham) split by social context (informal, formal) and report option (report, withhold).

Unlike memory reporting where report and withhold options cannot be analysed together, this is not the case with confidence ratings. Therefore, we ran an ad hoc exploratory analyses of confidence ratings (although the extant research does not allow for any specific a priori hypotheses regarding the impact of the TMS stimulation on confidence evaluations). See Fig. 4.

As, similar to the proportions analysis above, there was no difference between Active Control and Sham (*t*(36) = − 0.179, *p* = 0.859), we conducted a 2 Stimulation Group (Target, Active Control) X 2 Social Context (Formal, Informal) X 2 Answer Selection (Report, Withheld) mixed ANOVA on the confidence measures. There was a significant main effect of Answer Selection, *F*(1, 36) = 77.90, *p* < 0.001, η_p_^2^ = 0.443, showing that confidence ratings for reported answers (*M* = 34.86, *SD* = 15.64) were higher than those for withheld ones (*M* = 10.33, *SD* = 7.29). Stimulation Group and Social Context main effects were not significant (*F*(1, 36) = 0.641, *p* = 0.429 and *F*(1, 36) = 1.00, *p* = 0.324, respectively). More importantly, we found significant interactions between Answer Selection and Stimulation group (*F*(1, 36) = 4.67, *p* = 0.037, η_p_^2^ = 0.23), Answer Selection and Social Context (*F*(1, 36) = 77.98, *p* < 0.001, η_p_^2^ = 0.090), and the three-way interaction (*F*(1, 36) = 6.32, *p* = 0.017, η_p_^2^ = 0.008), although this was not the case for Social Context X Stimulation Group (*F*(1, 36) = 1.00, *p* = 0.324).

Our further analysis of the interaction between Answer Selection and Stimulation Group showed no differences when comparing the reported answers (*t*(36) = − 1.643, *p* = 0.109), and for the withheld one between the two groups (*t*(36) = 1.793, *p* = 0.081). In the follow-up analysis of the Answer Selection X Social Context interaction, we found that the confidence in the reported answers for the informal context (*M* = 40.37, *SD* = 10.81) was higher than for those reported in the formal context (*M* = 29.35, *SD* = 9.57), *t*(18) = 8.133, *p* < 0.001, *Cohen’s d* = 1.86. At the same time, for the withheld answers the confidence in the formal context (*M* = 15.85, *SD* = 7.13) was higher than in the informal context (*M* = 4.82, *SD* = 3.63), *t*(18) = − 8.133, *p* < 0.001, *Cohen’s d* = − 1.86. This result is interesting because it shows that only very low confidence answers were withheld in the informal context while this was not the case in the formal one.

Finally, the three-way interaction was carried by the absence of differences in confidence ratings between reported and withheld answers in the formal context within the Target stimulation group (*M*_*r*_ = 23.67, *SD* = 13.49; *M*_*w*_ = 19.43, *SD* = 11.52, *t*(18) = 1.059, *p* = 0.303) whereas the confidence for the reported answers was higher than for the withheld answers regardless of the Stimulation group or the Social context (for the informal context in the Target Stimulation: *M*_*r*_ = 37.94, *SD* = 17.36; *M*_*w*_ = 5.16, *SD* = 6.22, *t*(18) = 7.573, *p* < 0.001, *Cohen’s d* = 1.73; for the formal context in the Active Control Stimulation group, *M*_*r*_ = 35.02, *SD* = 15.93; *M*_*w*_ = 12.27, *SD* = 7.72, *t*(18) = 4.762, *p* < 0.001, *Cohen’s d* = 1.09; for the informal context in the Active Control Stimulation group, *M*_*r*_ = 42.80, *SD* = 15.76; *M*_*w*_ = 4.49, *SD* = 3.71, *t*(18) = 9.170, *p* < 0.001, *Cohen’s d* = 2.104).

### Gamma correlation

A one-way ANOVA on the gammas for the reported answers in the informal context showed no significant differences between the groups in the discrimination between correct and incorrect answers by the confidence ratings (gamma values for Target group: *M* = 0.91, *SE* = 0.11; Active Control: *M* = 0.91, *SE* = 0.09; Sham: *M* = 0.87, *SE* = 0.17); *F*(2, 54) = 0,525, *p* = 0.594).

## Discussion

The present study addressed, for the first time, causal involvement of the left middle frontal gyrus in communicational decisions in different social contexts. Our findings suggest selective involvement of the lMFG in formal social situations. As a result of the rTMS intervention in the lMFG function, we found that the pressure for reporting a certain amount of answers in the formal social context was mitigated, and the Target group participants reported significantly fewer answers in this context while no such effect was found in either the active TMS control or the sham control groups. This finding strengthens the previously suggested role of the lMFG in information control due to a perceived punishment or a pressure to behave in a particular manner. Also supporting our hypotheses, no differences were found in the distribution of the answers in the informal context in any of the three experimental groups. As expected, since this informal context lacks any type of incentive, punishment or pressure to follow any type of conventional behaviour, there is no need to involve the lMFG in the communicative decision process. That is, lMFG appears to play a role in communicative settings where the likelihood of negative outcomes depends on the information disclosed, but not in other situations.

Previous fMRI research linked the lMFG to the decision of withholding information, when encouraged by incentives to maintain accuracy high in memory reporting tasks^10^ and in the evaluation of punishment or a negative outcome^12^. Here, we further confirm these previous findings and extend this view to the broader case of conversational pragmatics. Previous research found different patterns of memory reporting in information exchange during conversational question–answer exchanges^2–4^. This behaviour could be explained theoretically by the perceived incentives that any given social situation conveys: there is the tendency to share any type of answer in informal contexts, whereas in formal contexts there seems to be certain contextual aspects (such as presenting ourselves as knowledgeable as possible^7^) that may lead to a higher propensity to withhold answers in case of uncertainty. The important novel finding in this study is that this “information-concealing” behaviour during social interaction may be *causally* linked to the lMFG, providing an important extension to the existing research in the field.

Our results are congruent with the notion of two systems of information processing involved in the decision-making process, dubbed System 1 and System 2^21^. This conceptualisation assumes that we might use System 1 to make heuristics-based and intuitive decisions while System 2 may preferentially be used when a more rational and thoughtful consideration is required, depending on the situation and the task. In an informal context (e.g., a chat with friends scenario used here), reporting a low-confidence answer (and thus running a higher risk of being incorrect) should not have particularly negative consequences. Indeed, in such situations even providing the wrong answer might just trigger a brainstorming session, leading to a joint discovery of the correct information.

In a more demanding formal context (the job interview model employed here), the stressful environment may tacitly motivate us to report only those answers, in whose accuracy we are considerably certain (these would be defined as the automatic answer, if conceptualised within System 1). However, if the questions are rather difficult and we are not certain of the answer, we may also report answers with lower confidence—simply because not reporting any answers for keeping the overall accuracy high (at the cost of informativeness) could make an overall negative impression^7,9,29^. In this context, irrespective of how many job interviews we have had, the requirement for cognitive resources remains high.

The use of Systems 1 and 2 in different social contexts motivates the expectation that rational thinking in an informal context/System 1 is rather limited; therefore, little or no differences in the pattern of answers regardless of the rTMS application was hypothesised, as it is not supposed to involve the prefrontal cortex to a high degree^30^. The opposite was hypothesised for the formal context/System 2 where the frontal lobe would be engaged under regular circumstances. As a result, we expected a higher rate of withheld than reported answers following lMFG inhibition, as the System 1 would be used in this context instead. Thus, another perspective on our results is conceptualising the aforementioned contexts as demanding low- vs. high-rationality.

Another report^31^ provides important additional support for our conclusions. In that study, participants completed a neuroeconomic task in the scanner with the aim to investigate the neurological basis of the Kahneman’s two-system decision-making framework. When participants’ choices were supported by System 1, the striatal areas were differentially activated, whereas when participants’ choices followed System 2 strategy, the frontal cortical areas were more active. Also, literature on cognitive control and prospective memory suggests a positive correlation between the degree of cognitive resources demanded by the task and the activation of the frontal cortical areas^32^, further reinforcing our conclusions.

The lMFG is among the brain areas implicated in executive functioning^14–20^ lMFG impairments have been associated with poor inhibition and with deficits in social communication skills^17–20^. This, among other factors, may result in inappropriate behaviour in social situations where communication exchanges take place. Yet, it is important to note that we do not claim that System 1 would completely “take the lead” when there is a disruption to the lMFG functioning. It has been shown, for example, that the left inferior frontal gyrus (lIFG) is one of the frontal areas that play crucial role in cognitive control, e.g., in the case of semantic and episodic memory. Our results, however, suggest that the modulatory effect of TMS on lMFG may include a social component since its disruption affected the memory reporting differently depending on the social context.

Still, further research on the neural basis of communicational exchanges in different contexts is necessary to strengthen our findings regarding differential involvement of the lMFG in the communicative decision-making process. While we registered important differences resulting from the contextual characterisation, it would be also important to study the role of lMFG in other social contexts, such as formal contexts that necessitate reporting answers (i.e., testifying in a court trial). The limited scope of the social contexts used here restricts the generalisability of the present findings, and further studies are certainly needed to explore a larger range of communicative situations.

Prefrontal cortex may be one of the key brain regions that differentiate humans from other animals; it has evolved in humans into a neuroanatomically rich and complex network, which supports a range of higher cognitive processes^33,34^. However, the detailed inventory of these cognitive processes remains unclear. To conclude, the current research provides new important knowledge regarding the nature of communicational exchanges mediated by different social contexts, and documents causal involvement of prefrontal cortex (lMFG in particular) in regulating associated task demands.

While, by showing a causal link between specific cortical area and communicative behaviour, this study offers a novel contribution to the young field of neuropragmatics, it is not without some limitations. First, the performance prior to the implementation of rTMS was not measured, therefore, it is unknown whether the baselines before the experiment differed between the groups. Still, participants were randomly assigned to each group, and the groups were matched on age, gender of other variables. Second, the reaction times were not collected (since the task involved typing and mouse cursor movements, rather that timed button-presses), so any potential effects of our experimental manipulations on response latencies could not be explored. Even though reaction-time recording is not usually done in memory-report studies, future research could explore whether there are differences in reaction times depending on the variables or conditions which could lead to important insights into the cognitive processes involved in such communicative decisions.

## Data Availability

Materials and data are available upon request from the first author.
